# Combined measurements of tumor number and size helps estimate the outcome of resection of Barcelona clinic liver cancer stage B hepatocellular carcinoma

**DOI:** 10.1186/s12893-016-0135-4

**Published:** 2016-04-19

**Authors:** Xin Wang, Zusen Wang, Liqun Wu

**Affiliations:** Department of Hepatobiliary and Pancreatic Surgery, Affiliated Hospital of Qingdao University, Qingdao, 266000 China; Jiangsu Road 16 Qingdao, 266000 China

**Keywords:** BCLC staging, Hepatocellular carcinoma, Prognosis, R0 surgical treatment, Risk factors

## Abstract

**Background:**

Although the Barcelona Clinic Liver Cancer (BCLC) staging system suggests that patients with stage B hepatocellular carcinoma (HCC) should be treated with transcatheter arterial chemoembolization instead of surgical treatment, recent studies indicated that the prognosis of surgical resection for patients with BCLC stage B HCC was better than that of TACE. However, the portion of patients with stage B that will achieve better outcomes from surgical treatment remains unclear. In this study, we identified risk factors that influence the prognosis of BCLC stage B HCC after R0 surgical resection to determine whether some patients with stage B HCC may benefit more from R0 resection than other patients and to provide a guideline to estimate the tendency.

**Methods:**

The clinical data of 78 patients with BCLC stage B HCC after R0 surgical treatment within 11 years were analyzed retrospectively, using relapse or death as the endpoint. Kaplan-Meier survival and Cox regression analyses were used to study prognosis (disease-free survival, DFS and overall survival, OS) and independent risk factors.

**Results:**

For all stage B patients, 1-, 2-, and 5-year DFS rates were 62.5, 36.4, and 16.6 %, respectively. Cumulative tumor size >5.0 cm and tumor number ≥4 were independent prognostic risk factors for DFS. The 1-, 2-, and 5- year DFS rates and OS rates of patients with at least one of these two factors were 49.0, 17.2, and 7.4 % (for DFS), and 78.6, 54.8, and 13.4 % (for OS), respectively, which were significantly lower than patients without these two factors (77.8, 58.3, and 27.2 % for DFS, and 94.4, 83.3,and 51.8 % for OS, respectively, *P* < 0.01).

**Conclusions:**

The analyses indicated that the outcomes of R0 resection were much better for patients with BCLC stage B HCC with two or three tumors and cumulative tumor sizes of ≤5.0 but >3.0 cm than other patients with stage B.

## Background

Hepatocellular carcinoma (HCC) is one of the five most common malignancies worldwide. Each year, more than 690,000 people die from liver cancer, and the number is increasing every year [1]. HCC is also the third most frequent cause of tumor-related death [2]. The clinical staging system of HCC has an important function for selecting treatment modes and evaluating prognosis, and in recent years, the Barcelona Clinic Liver Cancer (BCLC) staging system has received more attention. According to the BCLC staging system, only patients with very early stage and stage A (early stage) HCC should undergo surgical resection, ablation, or liver transplantation. Patients with stage B HCC should be treated with transcatheter arterial chemoembolization (TACE) [3]. However, many studies have indicated that for patients with BCLC stage B HCC, the prognosis of surgical resection is better than that of TACE [4–7], indicating that it is feasible and applicable for some patients with BCLC stage B HCC to undergo R0 section. However, the portion of patients with stage B that will achieve better outcomes from surgical treatment remains unclear. Moreover, the standard indicators or influencing risk factors for selection of HCC patients with stage B toward surgical resection have not yet been established. Therefore, study of the risk factors for prognosis of BCLC stage B after surgical treatment has profound significance in forming a guideline for clinical treatment of this sort of patients. Clarification of major factors influencing curative efficiency will enable clinicians to estimate whether a patient with BCLC stage B HCC may achieve a better prognosis from R0 resection and identification of those patients with non-improved outcomes should be more carefully monitored after resection. This study aims to provide surgeons with a guideline to estimate clinical treatment and predict prognosis to guide postoperative treatment.

## Methods

### Case data

From 2001 to 2012, 827 patients with HCC underwent R0 hepatic resection in the Affiliated Hospital of Qingdao University, China and 78 patients suffered BCLC stage B HCC disease. We declare that informed consents for the use of their clinical data in this study were obtained from all participants or their next of kin before follow up and before the study began. We thank them a lot for their consent of this study. BCLC staging was determined using liver ultrasonography or computed tomography (CT) and thorough clinical assessment. All the patients had 2–5 cancerous nodules with a cumulative tumor size of >3.0 cm, a Child-Pugh score of A-B, and performance status of 0, and thus, were classified as BCLC stage B. Pathological diagnoses were made for all patients. There were 66 men and 12 women (ratio 5.5:1) and their age ranged 33–80 years old, with an average of 55.9 years. The clinicopathological features of the 78 patients are shown in Table 1. The collection of clinical data was approved by the Ethic Committee of Affiliated Hospital of Qingdao University.

**Table 1:** Clinicopathological features of 78 patients with Barcelona Clinic Liver Cancer stage B hepatocellular carcinoma

Factors	Median (range or proportion)
Sex (male/female)	66:12 (84.6 %:15.4 %)
Age (years)	56.0 (33–80)
Alcohol consumption (no/yes)^a^	56:20 (73.7:26.3)
Preoperative TACE (no/yes)	67:11 (85.9 %:14.1 %)
HBsAg (negative/positive)	7:71 (9.0 %:91.0 %)
Anti-HCV (negative/positive)	76:2 (97.4 %:2.6 %)
ALT (U/L)	45.5 (12–191)
GGT (U/L)	49.5 (15–395)
Child–Pugh Classification (A/B)	76:2 (97.4 %:2.6 %)
Cirrhosis (no/yes)	5:73 (6.4 %:93.6 %)
Portal hypertension (no/yes)	65:13 (83.3 %:16.7 %)
Liver resection range (hepatic segment)	2 (1–4)
Resection margin (mm)^a^	5 (0–30)
Anatomical resection (yes/no)	18:60 (23.1 %:76.9 %)
Hepatic inflow occlusion (no/yes)	33:45 (42.3:57.7)
Intraoperative blood loss (mL)	300 (100–3500)
Blood transfusion (mL)	0.0 (0–1100)
Preoperative alpha-fetoprotein (μg/L)	17.8 (1.1–1211.0)
Cumulative tumor size (cm)	5.0 (2.5–20.0)
Tumor number	3 N
Differentiation (high/middle and low/necrosis)	3:73:2 (3.8 %:93.6 %:2.6 %)
Liver capsule invasion (no/yes)	12:66 (15.4 %:84.6 %)

*ALT* alanine aminotransferase, *GGT* gamma glutamyltransferase, *HBsAg* hepatitis B surface antigen

^a^Some patients lack data

### Surgical methods

All 78 cases underwent liver resection in accordance with the Couinaud segmentation method to implement hepatic segmentectomy or combined resection for adjacent liver segments (anatomical resection) or partial hepatectomy containing tumor (nonanatomical resection). R0 hepatic resection means there were no residual cancer cells present in the surgical margin when checked microscopically and no tumor visible with the naked eyes.

### Follow-up study

Serum alpha-fetoprotein, liver function tests, liver ultrasonography or CT and lung CT scan were performed monthly for 3 months after surgical resection. The time of recurrence after resection was adjudged by the presence of clear masses on imaging examination. The follow-up ended on 31 December 2015 or when patients died. The median follow-up time was 31.4 months (ranging 3.3–113.2 months).

### Statistical analysis

All the data were statistically analyzed with SPSS Statistics software for Windows, Version 13.0 (SPSS Inc., Chicago, IL, USA). The Kaplan-Meier survival analysis (log-rank test) was used to analyze disease-free survival (DFS) and overall survival (OS). The factors with *P* < 0.05 were enrolled in the Cox regression hazard model. *P* < 0.05 was considered significant.

## Results

### Risk factors for DFS and OS

Seventy-eight patients with BCLC stage B HCC underwent R0 hepatic resection. The OS rates for1, 2 and 5 years were 85.9, 67.9 and 29.8 %, respectively; the median OS was 35.0 months [95 % confidence interval (CI) = 29.4–40.6 months]. The DFS rates for 1, 2 and 5 years were 62.5, 36.4 and 16.6 %, respectively; and the median DFS was 15.0 months (95 % CI = 10.7–19.3 months).

In Table 2, we analyzed the relation of selected factors with the DFS with Kaplan-Meier analysis. The result showed that cumulative tumor size >5.0 cm and tumor number ≧4 were important risk factors that significantly influenced DFS (*P* < 0.01). Then the two factors were enrolled into Cox regression hazard model analysis which showed that the cumulative tumor size and tumor number were two significant independent risk factors for DFS (*P* < 0.05).

**Table 2:** Factors influencing disease-free survival (DFS) for patients with Barcelona Clinic Liver Cancer stage B hepatocellular carcinoma after R0 resection

Factors	Kaplan–Meier analysis		Cox regression hazard model	
	Median DFS (mo)	*P* value	HR(95 % CI)	*P* value
Sex (male/female)	15.0/13.0	0.771		
Age (≤60/>60 years)	15.0/13.0	0.530		
Alcohol consumption (no/yes)^a^	15.0/18.0	0.463		
Preoperative TACE (no/yes)	13.0/33.0	0.058		
HBsAg (negative/positive)	8.0/18.0	0.267		
Anti-HCV (negative/positive)	15.0/11.4	0.529		
ALT (≤50/>50 U/L)	23.0/11.4	0.076		
GGT (≤64/>64 U/L)	19.0/13.0	0.096		
Child–Pugh classification (A/B)	15.0/5.7	0.288		
Cirrhosis (no/yes)	25.0/15.0	0.317		
Portal hypertension (no/yes)	15.0/23.0	0.769		
Liver resection range (≤2/>2 segments)	17.0/8.0	0.084		
Resection margin (<5/≥5 mm)^a^	13.0/19.0	0.573		
Anatomical resection (yes/no)	14.6/15.0	0.783		
Hepatic inflow occlusion (no/yes)	13.0/19.0	0.223		
Intraoperative blood loss (≤1000/>100 mL)	18.0/13.0	0.282		
Blood transfusion (yes/no)	17.0/15.0	0.732		
Preoperative alpha-fetoprotein (≤20/>20 μg/L)	14.6/18.0	0.273		
Cumulative tumor size (≤5/>5 cm)	25.0/13.0	0.009	1.685 (1.011–2.808)	0.045
Tumor number (≤3/≥4)	23.0/10.1	0.000	2.681 (1.506–4.770)	0.001
Differentiation (high/middle and low/necrosis)	24.0/15.0/19.2	0.671		
Liver capsule invasion (no/yes)	13.0/17.0	0.882		

^a^Some patients lacked data

### Relationship between recurrence and tumor number

Patients with BCLC stage B HCC were divided into three groups according to tumor numbers which included 2, 3 and more than 4 tumors, respectively. Kaplan-Meier survival analysis was used to analyze the DFS for the three groups. The median DFS time and the DFS rate for1, 2 and 5 years decreased as tumor number increased (*P* < 0.01; Table 3).

**Table 3:** Disease-free survival of patients with different tumor number

No of tumors.	*n*	Median (months)	1 year (%)	2 years (%)	5 years (%)	*P*
2	39	28.8	76.9	53.8	23.1	0.001
3	17	19.0	58.8	25.3	22.1	
≥4	22	10.1	38.5	4.8	0.0	

**Fig. 1:**
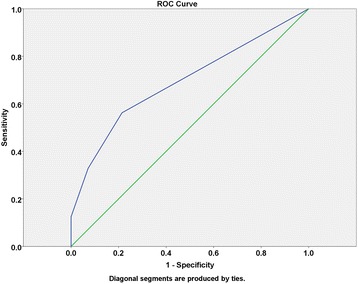
ROC curves of tumor number and recurrence

**Fig. 2:**
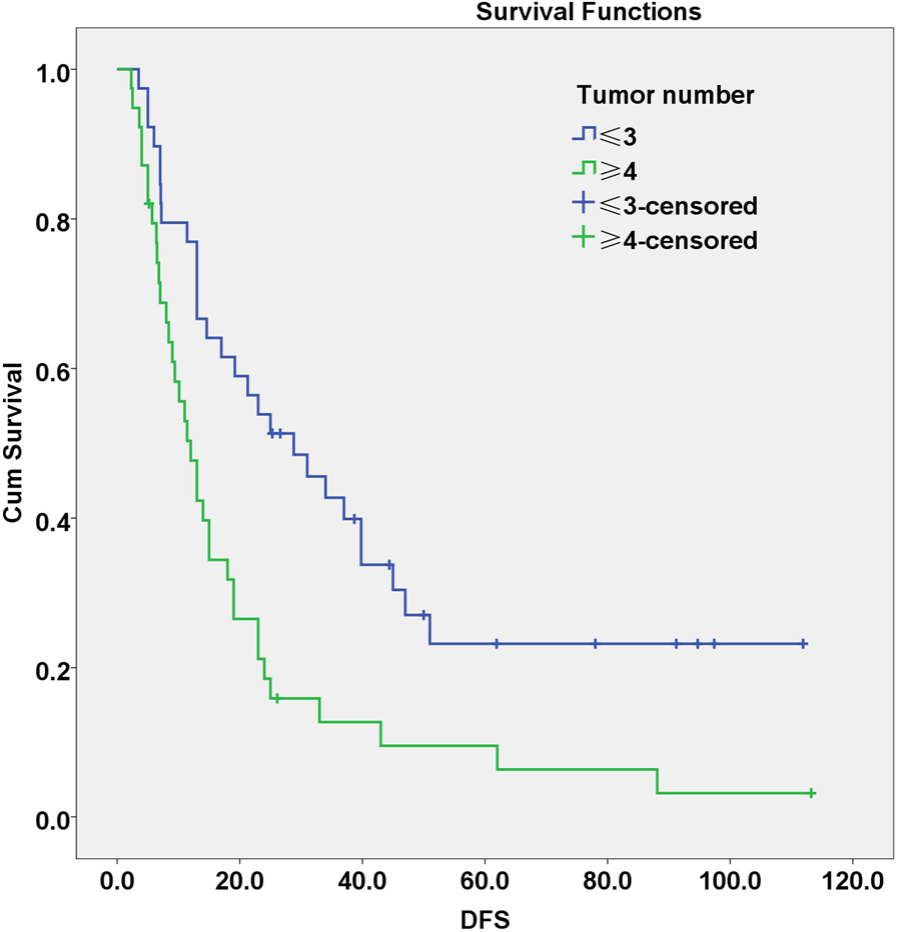
Disease-free survival (DFS) curves of tumor number ≤3 and ≥4. The upper curve represents DFS of patients with ≤3 tumors, while the lower curve represents DFS of patients with ≥4 tumors

Then, the receiver operating characteristic (ROC) curve was applied for further analysis. The tumor number was regarded as a test variable while the recurrence a state variable. The area under the curve was 69.4 % (*P* = 0.024) and the Youden index was 2.5 (specificity = 78.6 %, sensitivity = 56.3 %) (Fig. 1). We took the tumor number of 3 as the critical value according to the Youden index. The 1-, 2-, and 5-year DFS rates for the patients with tumor number ≤ 3 (56 cases) and ≥4 (22 cases) were 71.4, 48.2, and 23 % (for number ≤3), and 38.5, 4.8 and 0 % (for number ≥4), respectively (*P* = 0.000) (Fig. 2). These analytic data clearly indicate that the patients with 2 to 3 tumor number survived better with much lower recurrence rates than those with tumor number ≥4.

### Relationship between recurrence and tumor size

**Fig. 3:**
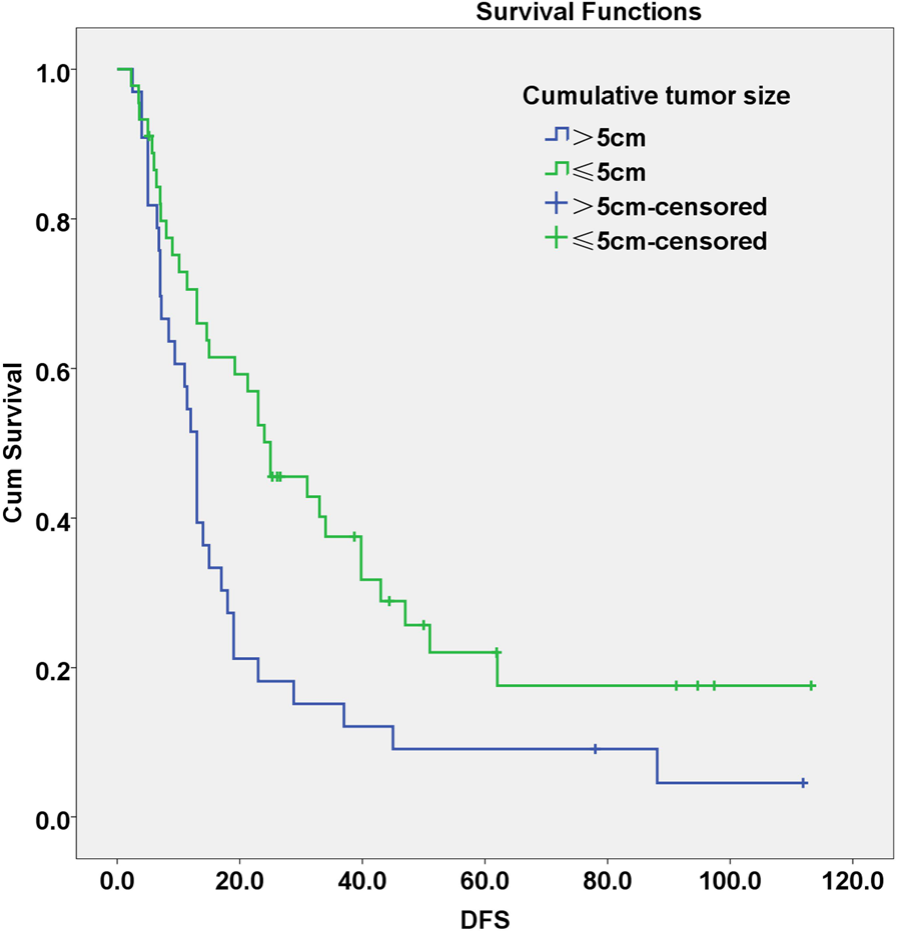
Disease-free survival (DFS) curves of cumulative tumor size ≤5 cm and >5 cm. The upper curve represents DFS of patients with a cumulative tumor size of ≤5.0 cm, while the lower curve represents DFS of patients with a cumulative tumor size >5.0 cm

On the basis of the cumulative tumor size, patients were divided into two groups, ≤5 cm (45 cases) and > 5 cm (33 cases). Kaplan-Meier analysis was performed to analyze the DFS for the patients in the two groups. The 1-, 2- and 5-year DFS rates were 70.6 , 50.1 and 22 % for the ≤5 cm group, and 51.5, 18.2 and 9.1 % for the > 5 cm group, respectively (*P* = 0.009, Fig. 3), indicating that the patients with cumulative tumor size ≤5 but >3 cm showed significant lower recurrence rates than those with cumulative tumor size > 5 cm.

### Comparison of DFS and OS between high-risk and non-high-risk groups

**Fig. 4:**
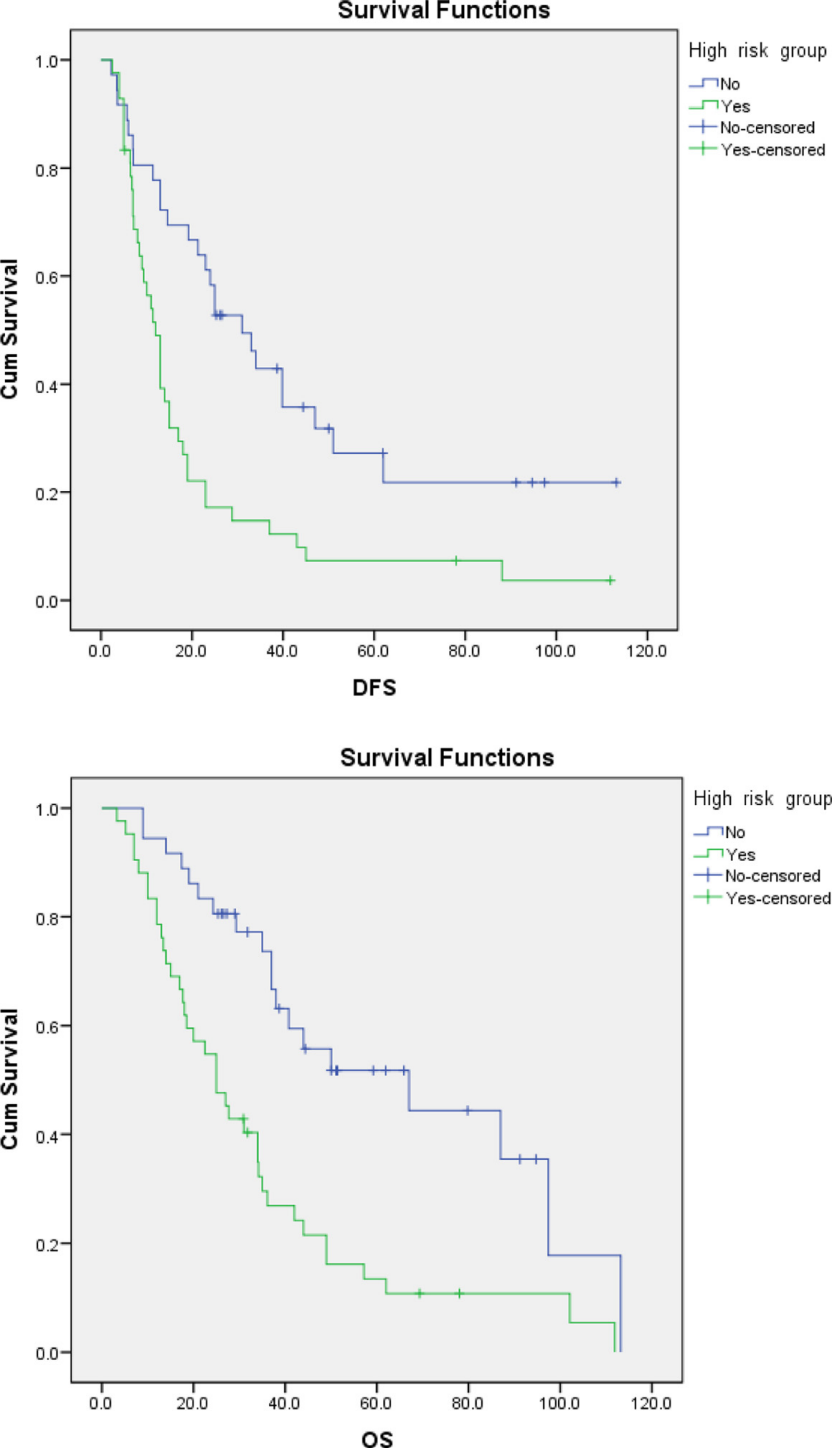
Disease-free survival (DFS) and overall survival (OS) curves of high-risk and non-high-risk group. The upper curves represent the DFS and OS of the non-high-risk group, while the lower curves are DFS and OS of the high-risk group

Patients with tumor number ≥4 and cumulative tumor size >5 cm were classified as the high-risk group (42 cases) while the other 36 patients as the non-high-risk group. Kaplan-Meier survival analysis was performed to analyze the DFS. For patients in the high-risk and non-high-risk groups, the 1-, 2-, and 5-year DFS rates were 49, 17.2 and 7.4 (for high-risk), and 77.8, 58.3 and 27.2 % (for non-high-risk), respectively (*P* = 0.001); the OS rates at 1, 2. and 5 years were 78.6, 54.8 and 13.4 % (for high-risk), and 94.4, 83.3 and 51.8 % (for non-high-risk), respectively (*P* = 0.000) (Fig. 4). The significance of the analytic results was in line with those in 3.2 and 3.3 sections, i.e., the recurrence rates of the non-high-risk group were significantly lower than those of the high-risk group.

## Discussion

Tumor recurrence after resection is an important factor affecting the prognosis of patients with HCC. The BCLC staging system was proposed in 1999 by Llovet et al. [8] and modified by the American Association for the Study of Liver Diseases in 2005 [9]. This staging system comprehensively considers tumor size and number, vascular invasion, liver function, and general condition. It has been accepted worldwide at present. The system recommends that patients with stage B HCC should be treated with TACE. However, many studies have indicated that for patients with BCLC stage B HCC, the prognosis for surgical resection treatment is superior to that for TACE. For example, from 171 patients with BCLC stage B HCC, Lin [5] found that the 1-, 2- and 3-year OS rates after surgical resection or TACE was 83, 62 and 49 % or 39, 5 and 2 %, respectively. The prognosis of surgical resection was obviously better than that of TACE.

Considering that patients with BCLC stage B HCC frequently have multiple tumors with or without vascular invasion and intrahepatic metastasis, we think that it is necessary to explore further the indicators of surgical resection for this stage HCC to obtain a better prognosis. In this study, we identified that tumor number ≥4 and cumulative tumor size >5 cm were independent risk factors influencing the prognosis of BCLC stage B HCC after R0 liver resection. Median DFS and OS in patients with these two factors were 12.0 and 25.0 months, respectively, which were significantly lower than those of patients with tumor number 2 or 3 and cumulative tumor size ≤5 cm (31.0 and 67.0 months, *P* < 0.01). In keeping with our current study, many studies have proved that tumor number [10–14] and tumor size [10, 11, 15–17] can affect prognosis after resection of HCC. For example, Shah et al. [10] discovered that multiple tumors and large tumor size are independent risk factors of postoperative recurrence of HCC.

It is clinically significant that tumor number and size have been identified as two independent risk factors. The blood supply of HCC is mainly from the hepatic artery and portal vein. Therefore, large tumors always have a more abundant blood supply, grow faster, break through the capsule more easily and transfer to and infiltrate the surrounding liver tissue readily. Large tumors also more easily cause invasion of the portal vein, increasing the probability of postoperative intrahepatic recurrence. Multiple tumors in the liver are mostly due to intrahepatic metastasis, and an increase in tumor number usually indicates more rapid metastasis. For these patients, even if the visible tumors are totally removed, small metastases may also be present in the liver, resulting in recurrence of HCC, which is consistent with the finding of this study that patients with HCC stage B with ≥4 tumors or a cumulative tumor size of >5.0 cm had higher postoperative recurrence rates.

Our study was limited by the small sample size and regional area, which may not be representative of patients with BCLC stage B HCC in other areas. Moreover, the follow-up period for some patients was relatively short. Nonetheless, these patients were included in the analysis because they meet the BCLC stage B-staging standard, although they had many significant risk factors reported already, e.g., age of >60 years; 4–5 nodules; tumor size of >5.0 cm; significant amount of perioperative blood loss; and much higher AFP, ALT, and GGT levels than normal. In spite of these limitations, the results of this study revealed that both tumor number and tumor size were the main staging indicators of BCLC stage B HCC. Therefore, our study has important significance in estimating clinical treatment and predicting the prognosis to guide postoperative treatment.

## Conclusions

This retrospective analysis suggests that patients with BCLC stage B HCC having two or three tumors and a cumulative tumor size of >3.0 cm but ≤5.0 cm achieved better outcomes from R0 surgical resection than other patients with stage B HCC.
